# Deficit schizophrenia: Concept and validity

**DOI:** 10.4103/0019-5545.39764

**Published:** 2008

**Authors:** Sandeep Grover, Parmanand Kulhara

**Affiliations:** Department of Psychiatry, Postgraduate Institute of Medical Education and Research, Chandigarh, India

First effort to distinguish and characterise psychotic illnesses was made by Emil Kraepelin,[1] who on the basis of observations of longitudinal course, categorised psychotic illnesses into ‘dementia praecox’ and ‘manic-depressive psychosis’. Many attempts were subsequently made to further characterise schizophrenia (dementia praecox), which culminated in the realisation that it is a heterogeneous disorder that has significant variability in clinical profiles, course and outcome. By 1980, to reduce the heterogeneity of this complex disorder, researchers tried to identify homogeneous subtypes in the hope to facilitate the identification of links between symptoms and putative neurobiological basis of aetiology. The division of symptoms as positive or negative and categorisation of schizophrenia as positive and negative subtypes[2] and type I and type II[3] became popular. However, researchers noticed that negative symptoms as described by Crow were not inherent to the disorder alone, but may also be due to neuroleptic medications, depression and environmental factors. This ushered in the concept of primary and secondary negative symptoms. To better understand primary negative symptoms and in a quest to homogenise a separate subtype of schizophrenia, Carpenter *et al.*[4] gave the concept of deficit and non-deficit schizophrenia.

## CONCEPT OF DEFICIT SCHIZOPHRENIA

According to Carpenter *et al.*,[4] the term ‘deficit symptoms’ should be used to refer specifically to those negative symptoms that are present as enduring traits. According to these authors, deficit symptoms may be present during and in-between episodes of exacerbation of positive symptoms. These deficit symptoms occur regardless of the patient's medication status and are not specifically responsive to anticholinergic drugs or antipsychotic drug withdrawal. It was further conceptualised that the presence of poor premorbid adjustment preceding initial psychotic episode may be manifestations of the deficit syndrome. In contrast, in non-deficit type of schizophrenia, negative symptoms may be present but demonstrate greater fluctuation, lack of persistence and temporal association with possible underlying causes like dysphoric states, drug status, etc. The patient with deficit symptoms may also have changes in symptom intensity and superimposed secondary negative symptoms, but a core of primary and enduring negative symptoms must be present. According to Carpenter *et al.*,[4] this approach to deficit symptoms requires clinical judgment based on longitudinal observation rather than a cross-sectional assessment to establish the presence or absence of the symptoms. To further stress their concept, they operationalised the diagnostic criteria for deficit schizophrenia, which is shown in Table 1.

**Table 1:** Criteria for schizophrenia with deficit syndrome[4]

The patient meets DSM-III criteria for schizophrenia At least two of the following negative symptoms are present: Restricted affect Diminished emotional range Poverty of speech with curbing of interest and decrease in curiosity Diminished sense of purpose Diminished social drive The negative symptoms are not fully accounted for by one more of the following: Depression or anxiety Durg effect Environmental deprivation Somet combination of two or more of the negative symptoms listed above has been present for the preceding 12 months and these symptoms were always present during periods of clinical stability (including chronic psychotic states) or during recovery from psychotic exacerbation. These symptoms may not be detectable during transient episodes of acute psychotic disorganization or decompensation

Carpenter *et al.* conceptualised that those patients who met only criterion 1 can be designated as *schizophrenia without negative symptoms*. Patients, who meet criteria 1 and 2 and possibly 4, but not criterion 3, can be designated as *schizophrenia with secondary negative symptoms*. Only those patients who satisfy all four criteria should be designated as *schizophrenia with deficit syndrome*. Those patients meeting criteria 1, 2 and 3, but not criterion 4, could either be schizophrenia with *primary, non-enduring negative symptoms* though these patients with passage of time may satisfy full criteria for schizophrenia with deficit syndrome and thus become *schizophrenia with deficit syndrome*.

To further distinguish deficit syndrome from primary, non-enduring negative symptoms, the authors[4] suggested certain conditions for those patients who meet criteria 1, 2 and 3, but not criterion 4 for schizophrenia with deficit syndrome. It was recommended that patients who had two or more prior periods of primary negative symptom (criteria 1, 2 and 3 for schizophrenia with deficit syndrome) followed by at least 3 months of remission of negative symptoms and where the current period of primary negative symptoms is of duration of 4 months or less should be considered to have primary deficit state. Later, ‘curbing of interests’ was added to the list of negative symptoms and it was specified that the negative symptoms must not be explicated by factors like anxiety, depression, drug effect, suspiciousness, formal thought disorder, hallucinations or delusions and mental retardation. To keep pace with nosological systems, the diagnostic criteria of schizophrenia were considered to be at par with the existing systems of DSM-III-R and DSM-IV. It was also noted that the deficit syndrome group was smaller than the group of patients with clinically significant negative symptoms as defined by rating scales such as the Scale for Assessment of Negative Symptoms, which rates both primary and secondary negative symptoms. In an attempt to further validate the deficit syndrome, an instrument, known as Schedule for the Deficit Syndrome (SDS) was devised.[5]

Over the years, considerable research on deficit schizophrenia has been conducted. Patients with deficit schizophrenia differ from non-deficit schizophrenia on variables such as risk factors, symptom profiles, neuropsychological functioning, family history, course of illness, treatment response and structural and functional neurobiology. Studies have also shown that deficit schizophrenia has long-term diagnostic stability.[6] Some researchers argue that deficit schizophrenia represents severe end of continuum of schizophrenia, whereas others argue that it represents a separate disorder/disease.

## VALIDITY OF DEFICIT SCHIZOPHRENIA

To address the question of validity of deficit schizophrenia, available data can be divided into three classes of potential validators. This scheme represents an adaptation and enlargement of the validating criteria for psychiatric illnesses as outlined by Robin and Guze.[7] The potential validators can be divided into *antecedent, concurrent and predictive validators.*

## ANTECEDENT VALIDATORS

### 

#### Demographic factors:

There is evidence to suggest that patients with deficit schizophrenia differ from non-deficit schizophrenia on sociodemographic variables pertaining to gender and marital status; on other sociodemographic variables no significant differences have been reported. In an initial report, Carpenter *et al.*[4] stated that patients of deficit schizophrenia were more frequently males; however, this finding was not replicated consistently. In a meta-analysis of 23 studies, Roy *et al.*[8] reported a significant association between male gender and deficit schizophrenia (pooled odds ratio: 1.75). The authors also stated that the association between male gender and deficit schizophrenia was not affected by breadth of diagnoses included and mean duration of illness. Studies have also shown that patients with deficit schizophrenia are less likely to marry before their first hospitalisation than patients with non-deficit schizophrenia, a difference that was still present even after controlling for age of onset.[9,10] Further, patients with deficit schizophrenia had an insidious onset more frequently.[10] However, some of the recent studies did not find any difference in marital status between deficit and non-deficit schizophrenia.[11] Some of the studies have also suggested that compared to non-deficit schizophrenia, patients with deficit schizophrenia have earlier age of onset.[11,12] In a follow-up study of patients after 15 years of first hospitalisation, Bottlender *et al.*[13] found that being male, single, longer duration of symptoms prior to first hospitalisation were the most powerful predictor of development of deficit syndrome during the follow-up.

#### Obstetric complications:

One study suggested that patients with deficit schizophrenia have lower rate of obstetric complications.[14]

#### Premorbid adjustment and functioning:

Studies have shown that patients with deficit schizophrenia have poorer premorbid adjustment than patients with non-deficit schizophrenia before the appearance of positive psychotic symptoms.[10–12,15–21]

#### Family studies:

There is some evidence to suggest that when compared with non-deficit schizophrenia, the patients with deficit schizophrenia appear to have an increased family history of schizophrenia,[14,22–24] with an odds ratio up to 3.45.[23] This higher risk in relatives persists even after correcting for sociodemographic and clinical variables.[23] However, there are contradictory findings regarding presence of other psychiatric disorders like depression, anxiety and alcohol use in the family.[14,22,24] The non-psychotic relatives of probands with deficit schizophrenia also show significantly more severe social isolation than the relatives of probands with non-deficit schizophrenia, in the absence of more severe depression or dysphoria and subclinical psychotic-like experiences (magical thinking). Studies have suggested that the deficit/non-deficit categorisation also has significant concordance within families, i.e. the presence of a sibling with deficit schizophrenia.[25–29]

#### Time of birth:

A winter birth excess has repeatedly been associated with schizophrenia. However, it is interesting to note that subjects with deficit schizophrenia demonstrate summer birth as a risk factor for the deficit form of schizophrenia. This finding has been consistently replicated in population-based studies of incident cases of psychosis[19,30] and prevalent cases studies.[31] Studies have also shown that this difference between the deficit and non-deficit schizophrenia groups remained significant after accounting for demographic characteristics and symptoms of disorganisation, hallucinations and delusions.[32]

## CONCURRENT VALIDATORS

### Clinical features

Clinical description of the concept of deficit schizophrenia has been described earlier. Various studies have evaluated the construct validity of deficit and non-deficit subtypes and have shown that deficit schizophrenia is associated with greater social and physical anhedonia,[20,33] more anergia,[20] less depression on self-report and by clinicians' ratings,[20,34,35] less suicidal ideation[10] and less severe delusions, content of which are exclusively social, such as delusions of jealousy[36] and poor insight.[24] Other clinical features, which distinguish deficit schizophrenia from non-deficit schizophrenia, include having less severe suspiciousness[16,17,36,37] and less substance abuse[17] and lesser awareness of dyskinetic movements.[15] During the stabilisation phase (3 months after hospital discharge) compared to non-deficit schizophrenia, deficit schizophrenia patients have lower depression, emotional alienation with loss of judgement, suspiciousness, obsessiveness, anxiety, social discomfort and social avoidance.[36]

#### Neurological examination:

On neurological examination compared to a matched non-deficit group, the deficit schizophrenia subjects were found to have higher level of soft neurological signs,[11] higher impairment in sensory integration, which was thought to be related to frontal and parietal function.[15,18]

#### Neuropsychological:

Patients with deficit schizophrenia exhibit selective impairments on a number of neurocognitive measures that are purportedly partly subserved by dorsolateral prefrontal basal ganglia-thalamocortical circuit. Buchanan *et al.*[38] compared subjects of deficit and non-deficit schizophrenia using the neuropsychological tests assessing the executive, visuospatial and memory functions, selected on the basis of their association with lesions of either the frontal, parietal or temporal lobes. They reported that patients with deficit schizophrenia showed significantly more impairment than non-deficit patients on two frontal lobe measures, the Stroop Color-Word Interference and Trails Making B tests and one parietal lobe measure, the Mooney Faces Closure Test. There were no differences in performance on the temporal lobe measures between the two groups.[38] In another study, patients with deficit schizophrenia performed poorly than patients with non-deficit schizophrenia and normal controls on continuous performance test (CPT) and span of apprehension task, whereas patients with non-deficit schizophrenia and normal controls did not differ from each other on CPT, but the non-deficit group also performed poorly on span of apprehension task compared to normals.[39] These differences between deficit and non-deficit groups were not related to total scores on thought, language and communication disturbances as measured by Brief Psychiatric Rating scale or to neuroleptic level.[39] Studies have also shown that, compared to patients with non-deficit schizophrenia, patients with deficit schizophrenia perform poorly on Wisconsin Card Sorting Test,[20,22,40] on some of items on visual memory but not on logical memory assessed by using Wechsler memory scale-revised.[20] However, no difference was noted in general intellectual functioning.[20]

#### Neurophysiological:

Oculomotor dysfunction, i.e. eye tracking disorder is significantly associated with deficit schizophrenia and it is suggested that both may have common pathophysiology of cerebral cortical-subcortical circuits.[41,42] Further, the eye tracking disorder in deficit schizophrenia has also been associated with poor sensory integration.[43] Functional imaging studies also suggest that deficit features and eye tracking dysfunction in deficit schizophrenia are due to abnormal function in overlapping brain regions (thalamic, frontal and parietal region).[44–46] Studies also suggest that deficit patients have generalised increase in visual reaction time[47] compared to non-deficit patients who had significant and abnormal asymmetry, with slower reaction time to targets presented in the right visual field than in the left visual field. Studies which have evaluated event-related potentials (ERPs) show that deficit schizophrenia and non-deficit schizophrenia groups differ significantly from each other and normal control group with regard to N1 amplitude (reduced in deficit schizophrenia) and topography (reduced current source density in cingulate and parahippocampal gyrus in deficit schizophrenia group), as well as P3 amplitude (reduced in non-deficit schizophrenia) and cortical sources (lateralised amplitude reduction over the left posterior regions and reduced current source density in left temporal and bilateral frontal, cingulate and parietal areas in non-deficit schizophrenia group). This suggests a double dissociation of ERPs, providing credence to the hypothesis that deficit schizophrenia represents a separate disease entity within schizophrenia.[48] One study evaluated prepulse inhibition (which reflects a measure of sensorimotor gating or filtering), which is a normal suppression of the startle reflex when intense startling stimulus is preceded by a barely detectable prepulse. It was found that patient with deficit schizophrenia has prepulse deficit in the 60 ms prepulse condition and a reduced facilitation in the 2000 ms prepulse condition; whereas prepulse inhibition in patients with non-deficit syndrome was impaired in the 240 ms prepulse condition.[49]

#### Neuroimaging:

There are inconsistent findings from structural imaging studies with regard to deficit schizophrenia. Some studies have reported that patients with deficit and non-deficit groups differ significantly in prefrontal white matter volume, with the non-deficit group having smaller volumes and the deficit group having similar white matter volume compared to normal controls.[50] Another MRI study reported that patients with negative symptoms have left temporal lobe parenchymal volume reduction, but this study did not examine prefrontal grey and white matter volumes separately and did not use the SDS to make the deficit/non-deficit diagnosis.[51] Positron emission tomography (PET) functional imaging studies, which have examined glucose and regional cerebral blood flow differences in patients with deficit and non-deficit schizophrenia, have suggested dorsolateral prefrontal cortex dysfunction responsible for production of deficit symptoms. Tamminga *et al.*[44] compared deficit schizophrenia with a non-deficit schizophrenia and normal controls and found decreased glucose use in the thalamus, frontal and parietal cortices in subjects with deficit schizophrenia. Both patient groups had decreased glucose use in the anterior cingulate and hippocampus. In another PET study[45] in which subjects were required to perform memory tasks, patients with deficit schizophrenia were characterised by decreased blood flow in the dorsolateral prefrontal and inferior parietal cortices, compared with the non-deficit group and normal controls.

During the attempt to retrieve poorly encoded words, patients of non-deficit syndrome had significantly greater activation of the left frontal cortex compared to patients with the deficit syndrome. However, there was no difference between the two groups in the activity of the hippocampus during memory retrieval.[52] Liddle *et al.*[53,54] reported that patients characterised by persistent negative symptoms had decreased resting regional cerebral blood flow in the dorsolateral prefrontal cortex. A proton magnetic resonance spectroscopy study showed significantly lower ratios of *N*-acetylaspartate (NAA) to creatine plus phosphocreatine in deficit schizophrenia compared to healthy subjects or non-deficit patients, a finding suggesting a neuronal loss in the medial prefrontal cortex of deficit patients.[55]

#### Viral markers:

Studies have suggested various biological markers/correlates of deficit schizophrenia. Waltrip *et al.*[56] reported significantly greater prevalence of antibodies to Borna disease virus in deficit schizophrenia compared to non-deficit schizophrenia. Another study suggested a link between the presence of Borna disease virus antibodies (p24 antibodies) and severity of negative syndrome in schizophrenia.[57] However, Kim *et al.*[58] did not find Borna disease virus transcripts in samples from any of the schizophrenic patients, i.e. belonging to deficit and non-deficit group. However, these contradictory results may be due to methodological problems.

Another study linked deficit schizophrenia with the presence of serum antibodies to cytomegalovirus (odds ratio = 2.01, *P* = 0.006), which remained significant even when the data were corrected for covariates like positive psychotic symptoms and demographic features known to be associated with cytomegalovirus seropositivity.[59]

#### Neurochemical:

There are conflicting results in relation to plasma homovanillic acid (pHVA) concentrations in deficit schizophrenia. Ribeyre *et al.*[60] reported that patients with deficit schizophrenia had lower pHVA concentrations than patients with non-deficit schizophrenia and none of the demographic, clinical and therapeutic variables could explain the difference. These findings were also reported by another study,[61] which in addition to lower pHVA concentrations found higher plasma 3-methoxy-4-hydroxyphenylglycol concentrations in the deficit compared with the non-deficit group even after controlling for demographic, clinical and therapeutic variables. However, these findings were contradicted by Nibuya *et al.*,[62] who found higher pHVA concentrations in deficit group compared to an age- and sex-matched non-deficit group.

Ozdemir *et al.*[63] measured NAA levels, which reflect neuronal density of the left frontal lobe in patients with deficit schizophrenia, and found that the patients with the deficit syndrome had significantly lower level of NAA than healthy subjects or non-deficit patients. In the same study, the patients with the deficit syndrome were found to have significantly lower ratios of choline compared with healthy subjects.

#### Genetics:

Studies which have evaluated the genetic differences between deficit and non-deficit schizophrenia fail to suggest differences between the two groups. Wonodi *et al.*[64] reported no significant difference in catechol-O-methyltransferase (COMT) allele or genotype frequencies between deficit and non-deficit cases, whereas there was significant difference in frequencies between schizophrenia cases (combined deficit/non-deficit) and healthy controls. One study, which evaluated association of neuregulin 1 gene (NRG1) assumed to be involved in glutamate neurotransmission and neurodevelopment (gliogenesis, neuronal migration and synaptic plasticity), found it to be associated with non-deficit schizophrenia, but not deficit schizophrenia, indicating a neurodevelopment cause in non-deficit schizophrenia, but not in deficit schizophrenia.[65]

## PREDICTIVE VALIDATORS

### 

#### Course and outcome:

Studies have shown that patients with deficit schizophrenia continue to exhibit poorer social and occupational function than other patients with chronic schizophrenia[10,16,17,21] and this difference cannot be attributed to more severe psychotic symptoms, demographic variables, greater anxiety, depressed mood or feelings of guilt[21] or substance abuse[16,17] in the deficit group. In long-term follow-up study (mean duration 19 years), patients with deficit schizophrenia display poor functioning compared with patients with non-deficit schizophrenia.[9,66] Some follow-up studies show that despite their poorer functioning, the patients of deficit group have a decreased severity of depressive mood and other dysphoric affect.[34,67] Studies have also shown that the deficit syndrome is highly stable[5,21] and was associated with very high risk of poor outcome, long-term disability, poor quality of life.[21,66]

#### Response to treatment:

Relatively few studies have attempted to examine the improvement of primary negative symptom with antipsychotics and even there the findings are conflicting. In clinical trials that used the SDS to distinguish deficit and non-deficit groups, the positive symptoms of patients with deficit schizophrenia showed the same therapeutic response to clozapine as patients with non-deficit schizophrenia, but there was no improvement in the negative symptoms of patients with deficit schizophrenia.[68] However, in the 1-year follow-up of the same subject, clozapine did not have any superior efficacy or long-term effect on primary or secondary negative symptoms.[69] Rosenheck *et al.*[70] failed to demonstrate any significant difference in response to clozapine between patients with high and low levels of negative symptoms at baseline or between patients with and without the deficit syndrome. One study that evaluated the role of psychosocial treatment in deficit syndrome, reported that negative symptoms of non-deficit schizophrenia showed improvement, but patients with deficit schizophrenia did not improve with social skill training;[71] however, the sample size in both the groups was very small (three patients each in both groups). In an open-label short-term trial (12 weeks) of olanzapine, patients who had non-deficit negative symptoms demonstrated improvements in positive and negative symptoms, level of functioning and extrapyramidal side effects over baseline. In fact, 35% of improvement in the non-deficit negative symptoms was accounted for by the improvements in positive symptoms, extrapyramidal symptoms and depression. In contrast, patients meeting criteria for the deficit syndrome improved significantly over baseline only in extrapyramidal side effects.[72]

## CONCLUSIONS

Criteria for the deficit group were explicitly developed by Carpenter *et al*.[4] Since the description of the criteria a lot of research has emerged, which has tried to distinguish deficit and non-deficit schizophrenia. However, it is to be noted that most of the studies have largely come from the Maryland group and their collaborators, which may raise question about the validity of the concept.

From available data, it can be concluded that there are meaningful and robust differences between deficit and non-deficit cohorts and the available data are consistent with both the separate disease hypothesis and the competing hypothesis of a disease severity continuum. Hence, further studies are required to firmly establish one or the other hypothesis. However, at present, it can be concluded that separating deficit and non-deficit groups reduces the heterogeneity of schizophrenia.
